# Protect AD: Realizing New Clinical Trial Possibilities

**DOI:** 10.1002/alz70857_103199

**Published:** 2025-12-25

**Authors:** Clive G Ballard, Ellie Pickering, Abbie Palmer, Millie Sander, Anne Corbett

**Affiliations:** ^1^ University of Exeter, Exeter, United Kingdom; ^2^ College of Medicine and Health, University of Exeter, Exeter, United Kingdom

## Abstract

**Background:**

The aim of PROTECT‐UK was to deliver an online ageing cohort for UK adults to facilitate large‐scale high quality longitudinal data collection and delivery of remote and hybrid clinical trials as an innovative cost‐effective low‐risk research solution.

**Method:**

PROTECT‐UK recruits adults over 40 in the UK through a website and app. All activities are completed online including annual neuropsychological assessment with the computerized FLAME battery, neuropsychiatric symptoms, physical and mental health and lifestyle data. DNA is collected remotely. Clinical trials are delivered within the study platform via full consent‐for‐contact and cohort pre‐screening which facilitates rapid recruitment. Participant engagement and retention is facilitated through access to evidence‐based cognitive training, regular communications and the opportunity to inform the research agenda.

**Result:**

PROTECT‐UK has collated data from 54,000 participants to date and has 30,000 active participants. International instances of PROTECT are live in Norway (*n* = 5000) and Canada (*n* = 2500). Participant retention in the UK is up to 80% year‐on‐year. 17,000 DNA samples have been collected.

PROTECT has recruited over 60,000 participants to online clinical trials, including large‐scale trials of cognitive training that demonstrated significant benefits and nutritional supplement trails including vitamin D supplementation in people with early cognitive deficits (*n* = 654), dietary nitrate in people with the ApoE4 gene (*n* = 120) and a hybrid trial of a Mediterranean Diet supplement involving a sub‐group recalled for venepuncture and physiological assessment (*n* = 150). Trials have achieved up to 95% retention and data completeness, with treatment compliance comparable to in‐person trials.

Genomic data has contributed to several published and ongoing studies including demonstrating increased schizophrenia and Alzheimer polygenic risk in people with mild behavioural impairment. Characterisation of the cohort shows there are >400 participants with GBA mutations and >800 E4 homozygotes with consent for contact.

**Conclusion:**

With proactive approaches, long term engagement can be achieved in digital platform studies. PROTECT has shown the feasibility of remotely running efficient and high quality clinical trials and collecting genomic and other biological materials to enhance research outputs.

